# Real-Time Evaluation of Breast Self-Examination Using Computer Vision

**DOI:** 10.1155/2014/924759

**Published:** 2014-11-11

**Authors:** Eman Mohammadi, Elmer P. Dadios, Laurence A. Gan Lim, Melvin K. Cabatuan, Raouf N. G. Naguib, Jose Maria C. Avila, Andreas Oikonomou

**Affiliations:** ^1^De La Salle University, 2401 Taft Avenue, Malate, Manila, 1004 Metro Manila, Philippines; ^2^BIOCORE Research and Consultancy International, Liverpool, UK; ^3^The University of the Philippines, Philippines; ^4^Nottingham Trent University, UK

## Abstract

Breast cancer is the most common cancer among women worldwide and breast self-examination (BSE) is considered as the most cost-effective approach for early breast cancer detection. The general objective of this paper is to design and develop a computer vision algorithm to evaluate the BSE performance in real-time. The first stage of the algorithm presents a method for detecting and tracking the nipples in frames while a woman performs BSE; the second stage presents a method for localizing the breast region and blocks of pixels related to palpation of the breast, and the third stage focuses on detecting the palpated blocks in the breast region. The palpated blocks are highlighted at the time of BSE performance. In a correct BSE performance, all blocks must be palpated, checked, and highlighted, respectively. If any abnormality, such as masses, is detected, then this must be reported to a doctor to confirm the presence of this abnormality and proceed to perform other confirmatory tests. The experimental results have shown that the BSE evaluation algorithm presented in this paper provides robust performance.

## 1. Introduction

Breast cancer (BC) is a major cause of cancer deaths among women worldwide [1, 2]. In 2010, BC killed around 425,000 women worldwide, and the trend is increasing [2]. It has been known that early detection of BC, coupled with proper treatment, increases the rate of survival [3]. Thus, proper education and appropriate BC screening methods, which are both effective and accessible to the general population, are necessary for solving this global problem, as suggested by the World Health Organization [3]. BC screening methods are mainly performed through breast imaging technologies, such as mammography [4] and visual/palpation-based physical examinations which include clinical breast examination (CBE) and breast self-examination (BSE) [4]. A combination of regular CBE, BSE, and counseling for breast cancer symptoms, beginning at the age of 20 years, coupled with annual mammography starting at the age of 40 years is recommended for early breast cancer detections [5, 6]. BSE has the advantages of being simple, inexpensive, and non-invasive [7, 8]. This is ideal for breast cancer awareness and screening in resource-limited areas of developing countries where women cannot afford annual medical examination (CBE) and mammography [9]. Furthermore, BSE has been shown to detect new breast cancers (interval cancers) in high-risk women subjected to regular CBE and mammography [10]. Additionally, mammography is not accurate on dense breast tissues common to younger women aged below 40 [10]. Finally, it is hypothesized that a woman would be highly sensitive to changes in her own breast tissues by undertaking a regular BSE as an important part of breast cancer detection [7]. Women, who perform BSE, should receive proper training/instructions and have their technique supervised and regularly reviewed. Thus, the development of an application that can guide and evaluate BSE performance objectively is very significant. In this paper, an algorithm is presented as a part of the computer vision system for evaluating the actual BSE procedure. Although independent research on the application of computer vision to BSE exists [11, 12], to the best of our knowledge, an integrated computer vision-based BSE system that can evaluate the user's performance has never been developed.

The paper is organized as follows: the computer vision-based approach to breast self-examination is discussed in Section 2, the datasets are explained in Section 3, the nipple detection and tracking is presented in Section 4, while left and right breast regions localizing and tracking are explained in Section 5, the palpation detection approach is discussed in Section 6, and the experimental results are stated in Section 7. Finally, the paper is concluded in Section 8.

## 2. Intelligent Computer Vision-Based Breast Self-Examination (ICBSE)

Breast self-examination mainly consists of two stages: visual and tactile. Visual examination searches for abnormal changes in the breast such as dimpling of the skin, changes in breast contour, inversion of the nipple, and any change in the size, shape, and symmetry, while tactile examination checks for any new tangible abnormality in the breast [3, 13]. The tactile examination must be performed by checking and palpating the entire breast region for detecting abnormalities.

**Figure 1:**
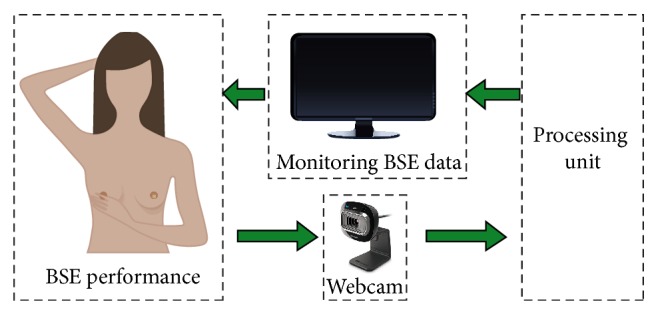
Intelligent computer vision-based breast self-examination (ICBSE).

The main goal of ICBSE is to monitor and evaluate the BSE performance in terms of covering and palpating the entire breast region. The breast region measures the totality of examination in terms of coverage area, from the armpit region, down to the lower part of the breast tissue, and upward towards the collarbone [3]. As shown in Figure 1, ICBSE employs a camera to capture the video of an actual BSE. Then, the video is analyzed in the processing unit for the evaluation of the BSE performance. Finally, the evaluated results are monitored on the screen in real-time.

**Figure 2:**
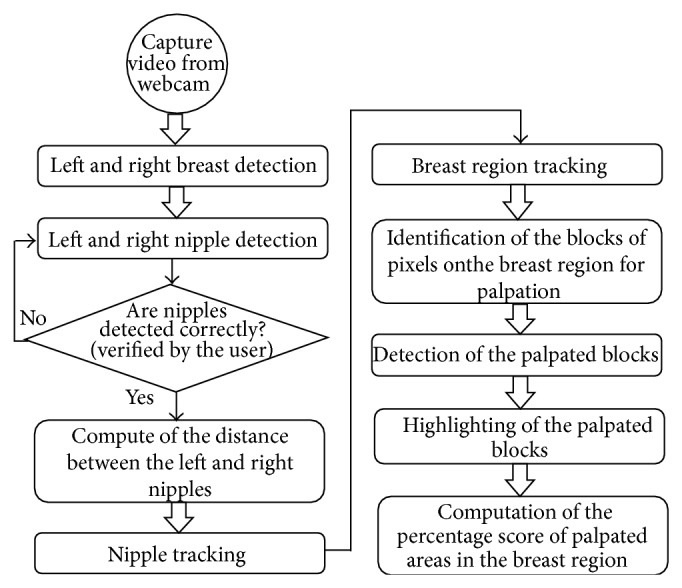
The block diagram of the algorithm for the BSE evaluation.

The video processing module in the ICBSE is concerned with the following objectives: nipple detection and tracking; breast region identification and tracking; palpation detection on the breast region; and monitoring the evaluated results. The block diagram of the video processing module in the ICBSE, which is expressed in this paper in detail, is shown in Figure 2.

## 3. Image and Video Datasets

There are mainly 4 datasets were used in this study. For simplicity, these datasets are called Dataset-1, Dataset-2, Dataset-3, and Dataset-4. The first two datasets were used to train and test the breast and nipple detection approaches using cascaded AdaBoost and neural network. The third and fourth datasets were used to just evaluate the nipple tracking and palpation detection approaches and they were not used for any training.

### 3.1. Dataset-1

Dataset-1 consists of positive images including the breasts and negative images without any breast for training and testing of breast detection using the cascaded Adaboost classifier. The number of positive samples in this dataset is 1,000 and the number of negative samples is 8,000. The negative samples were downloaded from [14]. 700 positive samples were downloaded from [15] and 300 positive samples were captured by the authors from women volunteers. The positive samples were selected and captured with different forms of breasts and luminance conditions. Since, in the actual BSE performance, the women must sit in front of the webcam and manipulate the computer, cellphone, or tablet, the distance between the woman and the webcam would be in the range of 40–80 cm. Thus, in this dataset, the positive samples were captured and selected with the mentioned distance between the webcam and women. All positive and negative samples were resized to 320 by 240 pixels.

### 3.2. Dataset-2

Dataset-2 consists of positive and negative samples for training and testing the neural network for the nipple detection. The positive samples are those subwindows containing the nipple, and the negative samples are those subwindows without any nipple. The positive samples were captured in different luminance conditions, with different types and colors of nipples. In Dataset-2, the number of positive subwindows is 250, and the number of negative subwindows is 5,600. All positive and negative samples were resized to 10 by 10 pixels.

### 3.3. Dataset-3

Dataset-3 consists of 20 videos for the evaluation of the nipple tracking algorithm. The length of the videos is 10 seconds, including 200 frames. For the evaluation, 40 frames are extracted from each video (1 frame from each 5 frames). Thus, in this database, the total number of frames is 800 for the evaluation of nipple tracking. The videos were captured in different luminance conditions, including shadowing and occlusion on the nipples.

### 3.4. Dataset-4

Dataset-4 consists of positive and negative videos for the evaluation of the palpation detection algorithm. Since the appropriate time for palpation on each block was considered to be 10 seconds, based on the pathologist's suggestion, the length of the videos in Dataset-4 is 10 seconds. The positive videos are those with correct palpation on the block and the negative videos are those without any palpation on the block. The number of positive videos is 20 and the number of negative videos is 30. The sample videos were captured in different luminance conditions. The videos were captured by the researcher from women volunteers.

## 4. Nipple Detection and Tracking

This section describes the nipple detection and tracking procedure. In the nipple detection algorithm, the left and right breasts are detected using the Haar-like features and cascaded Adaboost classifier based on the algorithm stated in [16]. Then, the features and pixel intensities inside the breast regions are analyzed for detecting the nipples. The integral imaging [16, 17], color features [18], and neural network [19, 20] are used for nipple detection. Then, the detected nipples are captured, saved, and used for tracking using template matching method [21, 22].

### 4.1. Breasts Detection Using Haar-Like Features and Cascaded AdaBoost Classifier

For a known training data set and set of features, every machine learning method can be used in learning a classification. Boosting is a kind of machine learning technique which performs supervised learning [23]. The concept is that a strong classifier can be created by linearly linking a number of weak classifiers. Weak classifiers are acquired by response of the features from the training data and have a detection rate which is slightly improved than the random guessing results [16]. To increase the detection rate, it is vital to employ a machine learning approach. AdaBoost is the most popular boosting algorithm, which was introduced by [24]. In this research, the numbers of negative and positive samples are 8,000 and 1,000, respectively. The size of the window for locating the features is 24 by 24 pixels. Cascade of classifiers is used to increase the accuracy and speed of the algorithm [16]. As a substitute of applying all the features on a window, the features are grouped into different stages of classifiers for one-by-one application [16]. In this study, the complete breast detection cascade has 7 stages. A Gaussian filter was applied on all the samples before starting to train. The reason of using Gaussian is to reduce noise and smooth the images [25]. Here, the size of the Gaussian kernel is 3 by 3 pixels and the sigma was considered to be 0.3 based on the kernel size. The detected left and right breasts are used as the regions of interest (ROIs) for nipple detection.

### 4.2. Feature Extraction for Nipple Detection

**Figure 3:**
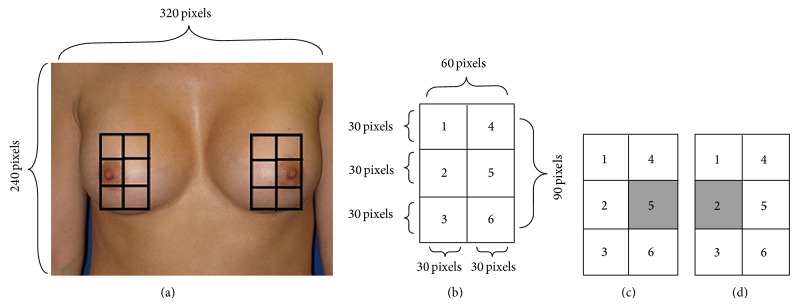
(a), (b) The structure of the ROI windows. (c) The sum of the pixels in 5th square must be less than others for left-nipple detection. (d) The sum of the pixels in 2nd square must be less than others for right-nipple detection.

The extracted ROIs (left and right detected Breasts in the previous section) are first resized to 200 by 200 pixels. The red component is used for the nipple detection. The reason for using the red level is that the color of nipples is close to red, and the result would be more accurate while analyzing pixel intensities for detecting the nipples. Then, histogram equalization is applied on the frames. The reason for using histogram equalization is to enhance and adjust the contrast in the detected breast frames. The cumulative histogram equalization was carefully chosen due to its simple and effective performance in the C language [26]. Subsequently, image normalization is performed on the frames. This process should be employed for decreasing the effect of luminance conditions on the nipple detection algorithm. At the next step, integral imaging is performed on the frames. The integral image is used as a fast and effective way of calculating the sum of pixel intensities in a given image. The integral image features are accomplished by means of convolution between a kernel and breasts ROIs. The kernel size is 60 by 90 pixels. The form of the kernel is shown in Figure 3. Here, the kernel window has six smaller windows (subwindows) inside it. The size of each subwindow is 30 by 30 pixels. The sums of the pixel intensities, in the subwindows, are computed for detecting the nipples. Since the color of the nipples is darker than their surrounding areas, the sum of the pixel intensities in the nipple area is smaller than the sum of the pixel intensities in the surrounding areas. The rules for detecting the nipples ROIs are described below.

- If the sum of the pixel intensities in the fifth subwindow is smaller than in other subwindows, the fifth subwindow is considered as an ROI for detecting the left nipple.
- If the sum of the pixel intensities in the second subwindow is smaller than in other subwindows, the second subwindow is considered as an ROI for detecting the right nipple.

Using this method, the nipples are detected. However, there might be several false positive detections. To eliminate the false nipple detections after applying integral imaging on the breasts ROIs, the color features and pixel values of the nipple ROIs (second or fifth subwindow in the kernel) are extracted and applied in the neural network to classify the nipple and nonnipples ROIs. In this study, the color features and gray pixel values are used for constructing the feature vector. The kernel subwindows are resized to 10 by 10 pixels prior to using the gray pixel values for the classification. After performing several experiments, this size was selected since it yields the most accurate result in classification. The color features are the mean and standard deviation of the pixels in the resized subwindows. As described in [27], the RGB model is not appropriate for describing a color image from a human-perception point of view. The hue, saturation, and value (HSV) model is more appropriate for the human visual system to define a color image. Since the human visual system perceives the color object, the HSV color space is proposed to compute the color moments [27]. The color moments are only calculated in hue level because the hue level defines the color appearance and is more effective in judging perceptual resemblance. Therefore, the feature vector for using in the neural network has 102 elements (100 elements from the pixel values and 2 elements from the color features).

### 4.3. Neural Network for the Nipple Detection

**Figure 4:**
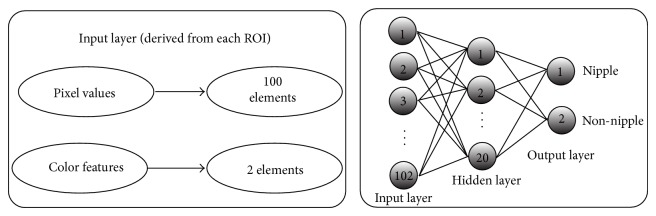
The structure of the neural network for nipple detection.

Artificial neural networks (ANNs) have been employed for pattern classification and recognition in different applications [19, 20]. The multilayer perceptron neural network is among the best neural networks in manipulating classification problems. These networks consist of 3 layers in most cases, input, hidden, and output layer. Generally, the ANN has a number of activation functions, training methods, and variations. The activation function of the hidden and output layers has to be adapted in accordance with the problem. In this research, the activation function is sigmoid. The optimization methods to minimize error are different but Levenberg-Marquardt has shown to have better results in most cases and therefore are used to train the neural network. Training continues until the network completes the modification of the validation set. As mentioned previously, the feature vectors with 102 elements are used for training and testing the neural network. The ANN has 102 neurons in the input layer, 20 neurons in the hidden layer, and 2 neurons in the output layer. All the elements are normalized to the range [0 ⋯ 1] prior to training and testing the ANN. The ANN structure used in this study is shown in Figure 4.

250 positive and 5,600 negative subwindows were used for training and testing the neural network. Positive ROIs are those subwindows including a nipple, and negative ROIs are those subwindows without a nipple. There are two neurons located in the output of the neural network. One is for classifying the nipples, while the other is for classifying a bounding window without a nipple. Following preprocessing and feature extraction, the vectors of features are used as the input for training the neural network. 80% of the data was used for training, 10% for testing, and the remaining 10% for validation. The test set provides a completely independent measure of network accuracy. Since the ANN starts with random initial weights, the results differ slightly every time the ANN is run. The stopping criterion for the training is based on the fluctuation of validation. During iterative training of the ANN, an epoch is a single pass through the entire training set, followed by testing of the verification set. The training stops based on the constant error values of validation at epochs 226, 227, and 228. The best performance as measured in terms of mean squared error was 0.0001205 after 228 epochs with a 98.7% correct classification accuracy. After detection of the nipples and verification of correct detection by the user (woman who performs BSE) the left and right nipples are captured and saved as two JPEG files for using in the tracking process.

### 4.4. Nipple Tracking

Template matching is used for tracking the nipples. The references of left and right nipples for template matching are derived from the detected nipples in the previous section. For nipple tracking, the normed square difference matching method (NSDTM), normed correlation matching method (NCTM), and normed correlation coefficient matching method (NCCOEF) were tested to recognize the most accurate model. The testing process is described in Section 7. The NCCOEF was considered as the most accurate method for tracking the nipples. This method matches a template relative to its mean against the image relative to its mean, so a flawless match will be 1 and a perfect mismatch will be −1; a value of 0 simply means that there is no correlation [21, 22].

## 5. Breast Region Localizing and Tracking

**Figure 5:**
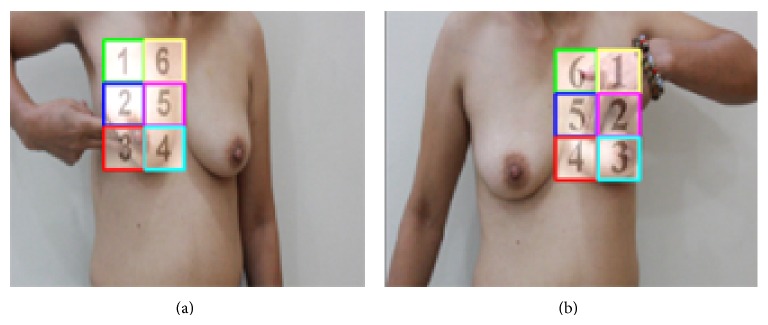
The samples of breast regions identification.

BSE should be performed by checking the entire breast region for detecting the lumps and/or abnormalities. Therefore, the breast region must be properly detected for an objective evaluation of BSE performance. Each breast region is identified by a matrix of size 2 by 3. Here, each small window in the matrix is called a block. The blocks are shown on the screen and women should perform palpation and checking the breasts by following the numbers of the blocks.  Figure 5 shows the samples of the left and right breast regions identification.

**Figure 6:**
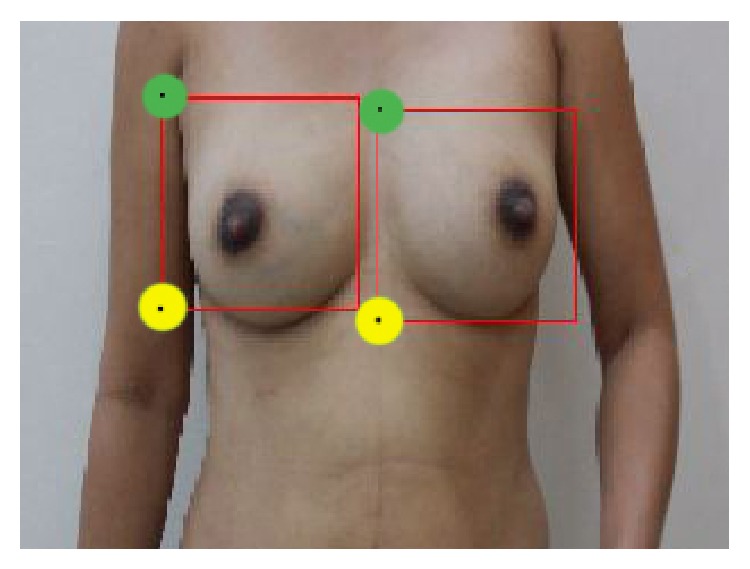
The detected breasts with starting and ending points.

Following the detection of the nipples in the first section, the distance between the nipples is calculated by counting the pixels. There is a need to compute the distance between the nipples for locating the matrix on the breast regions while a woman performs BSE. In order to track the left breast region, the right nipple is used as the reference, and to track the right breast region, the left nipple is used as the reference. The reason for using the opposite nipple as the reference in tracking the breast region matrix is that the breast is a nonrigid organ and it “moves” while palpation is performed on it. However, the opposite breast is practically stationary. The matrix is located on each breast region based on the distance between the nipples. In this algorithm, the point at the top and left side of the square of breast detection, using cascaded AdaBoost in the previous section, is called the starting point, and the point at the bottom and left side of the square is called the ending point. In Figure 6, the starting and ending points are shown by green and yellow colors, respectively.

The width of the matrix is based on the distance between the nipples. It is computed by dividing the distance between the nipples by two, and it starts from the starting point. The length of the matrix is based on the distance between the starting and ending points. Since the motion in the *Z* axis is very small at the time of BSE performance (there is no zoom in or zoom out), the algorithm does not need to compute the distances in real time. Instead, they are computed in the first step and the BSE performance is started after verifying the correction of breasts and nipples detection by the user.

Since the BSE procedure is normally carried out using the left hand for the left breast and the right hand for the right breast, occlusion and coverage of the opposite nipples do not occur during it. However, in order to enhance robustness, a simple method for time of occlusion is included here. The rules for the mentioned method are described below.

- If the distance between the starting points of the matrices on two frames is over 20 pixels, this is considered as a fake motion and the new position of the matrix would be based on data from the previous frame.
- If the distance between the starting points of the matrices on two frames is less than 20 pixels, this is considered as a real motion and the position of the matrix would be based on data from the new frame.

## 6. Detection the Palpated Blocks

The detected blocks in the previous section must be palpated in order to identify abnormalities. This section explains how the palpated blocks are detected in real time.

There are six blocks in each matrix for the left and right breast regions. Thus, the palpation and checking of the left and right breast regions has six stages, respectively. The palpation detection for each block is derived from the motion detection using the optical flow algorithm. There are many methods available to calculate optical flow, but the goal is always the same: for every pixel the velocity vector is calculated [28]. Originally, optical flow algorithms used gradient-based estimation. The results provided are acceptable only for certain classes of motion [29]. Therefore, the palpation detection procedure calculates optical flow through the tensor-based algorithm, proposed by Farnebäck in [30, 31]. The Farnebäck model is a dense optical flow algorithm. The term dense means the motion is calculated for every pixel in the block. This is usually costly, but Farnebäck's model is linear which can easily be solved. Farnebäck's method uses polynomial expansion to approximate the neighbors of a pixel [31]. The expansion could be perceived as a quadratic equation with matrices and vectors as variables and coefficients. This dense optical flow yields a motion field from two successive video frames [31]. The notion is to signify the image signal in the neighborhood of each pixel by a 3D surface and determine optical flow by finding whether the surface has moved in the next frame. The optimization is not performed on a pixel-level basis but rather on neighborhood-level, so that the optimum displacement is found for both the pixel and its neighbors [31].

While extracting each block containing palpation information, the optical flow is computed. Each block is resized to 100 by 100 pixels prior to applying optical flow. For the Farnebäck algorithm, three parameters are adjusted to detect palpation: the averaging window size, the size of the pixel neighborhood that is considered when finding the polynomial expansion in each pixel, and the standard deviation of the Gaussian used to smooth derivatives in the polynomial expansion. The remaining parameters are set to their default OpenCV values. The averaging over neighborhoods is carried out using a 70 by 70 Gaussian weighting function with standard deviation 6. The polynomial expansion is conducted with a 19 by 19 Gaussian applicability with standard deviation 3.5. In order to reduce the errors near the borders, the polynomial expansions have been calculated with certainty set to zero on the border. Furthermore, pixels close to the borders have been given a reduced weight since the expansion coefficients can still be expected to be less reliable there.

Finally, a motion vector is determined for each small neighborhood. Initially, it was intended to exploit the data describing the directions of the movements but this information seemed to be too noisy and unstable in actual tests. Consequently, it was decided to use only the information about the velocity; that is, only the length of each motion vector is considered.

**Figure 7:**
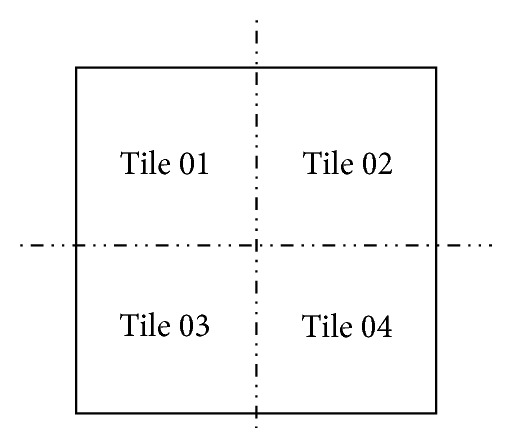
Dividing each block into four tiles.

**Figure 8:**
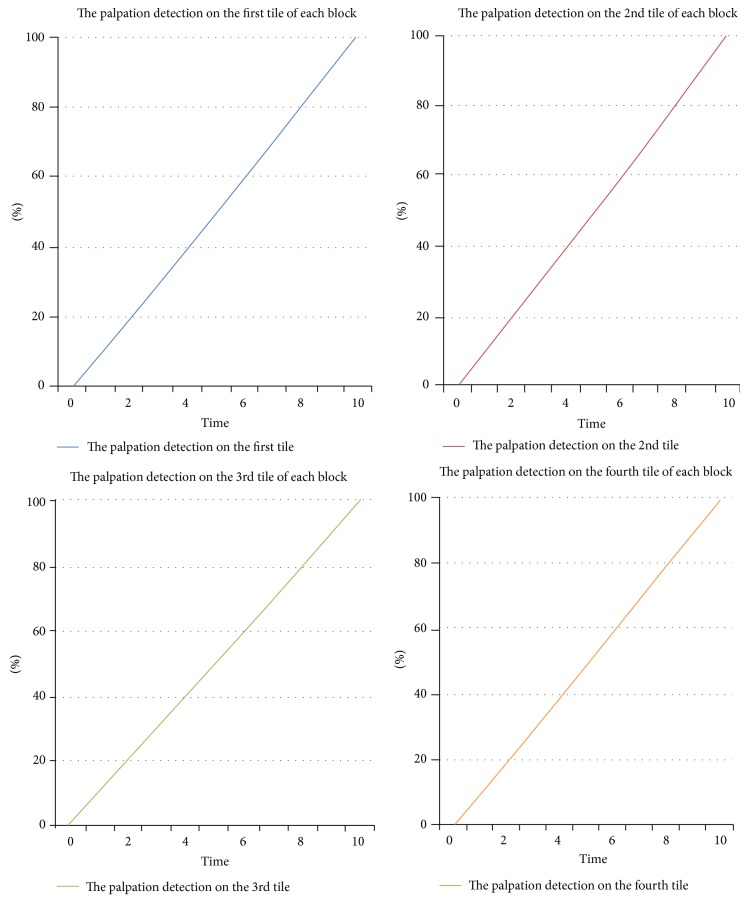
The palpation detection on the tiles of a block.

In order to increase the accuracy of the palpation detection algorithm, the output matrix of the optical flow is divided into four equal tiles as shown in Figure 7. Since, sometimes, the palpation is performed on the borders of the blocks, there is a need to detect palpation for each tile separately. Generally, it is not possible to divide the blocks before applying the optical flow due to the size of the blocks being very small and the Farnebäck algorithm with the stated parameters not able to detect palpation, based on the experiments. After dividing the output matrix of optical flow into four equal tiles, the NormL2 is applied in each tile to compute the integer for each tile. Using the threshold on the NormL2 value and the time, the palpation on each tile and block is detected. The most efficient threshold was considered 100,000, based on the experimental results. The approximate time for the palpation is 10 seconds based on the suggestions of the pathologists. Since the minimum fps is 20 in this research, there must be a minimum of 200 frames for each block to consider a palpation. Generally, the motion of the fingers on each block is considered as the correct palpation using the stated method.  Figure 8 shows the graphs relating to the percentage of palpated tiles with respect to time.

As shown in Figure 8, if the palpation is performed correctly with respect to time and the threshold on the NormL2 value, the percentage score for palpating the tiles gradually increases until it reaches 100%. This process is carried out for each tile in each block separately. However, if palpation is undertaken on four tiles simultaneously, the percentage scores of four tiles would increase simultaneously. But, if at the time of palpation on each block, the next block is palpated, the palpation on the next block is ignored; that is, the palpation process must be performed for the blocks separately and sequentially.

**Figure 9:**
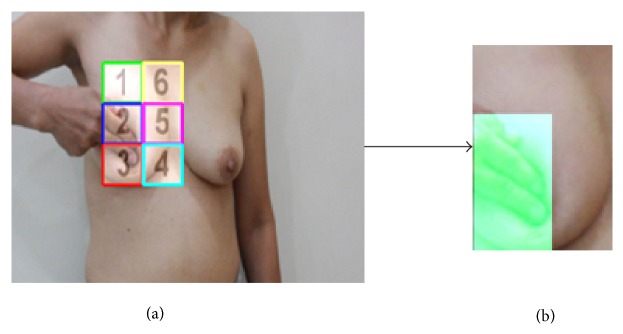
(a) The sample of BSE performance. (b) The 2nd and 3rd blocks were highlighted after palpation.

Each block is highlighted after performing palpation. This process is employed to monitor the BSE evaluated results to the user (woman who performs BSE). Thus, the user continues palpation until all blocks are highlighted. This procedure is performed for the left and right breasts separately. The palpation percentage score is also monitored in real time. This score shows the actual breast region being palpated. The sample of highlighted blocks is shown in Figure 9.

As shown in Figure 9, the second and third blocks are highlighted, whereas the remainders of the blocks still need to be palpated and checked.

## 7. Experimental Results and Evaluations

### 7.1. Performance Criteria

Since a limited number of datasets are available, cross validation is an appropriate method to evaluate the performance of the Cascaded Adaboost and Neural Network models. The holdout method is the simplest method of cross validation [32]. The data set is divided into two sets, which are called the training and testing sets. The learning algorithms decide the classifier using the training set; then, the classifier computes the values for the data in the testing set. It is vital that the classifier does not use the test dataset during the training process. The errors they make are accumulated as before to give the mean absolute test set error, which is used to evaluate the model. The holdout method is usually preferable to the other cross validation methods since the number of repetitions to evaluate the learning algorithm is zero [32].

The evaluation of nipple and breast region detection is carried out over the image subwindows. For this purpose the comparison of the ground truth data with the result of the cascaded Adaboost and neural network for each image is required. The performance equations are the key concept to evaluation of the algorithm and defined by labeled ground truth data and the result made by the classifiers. The most popular method is confusion matrix [33]. It contains information about actual and predicted classifications conducted by a classification system. Performance of such systems is normally evaluated using the data in the matrix. In this paper, the confusion matrix is also used for the evaluation of the palpation detection algorithm based on the positive and negative videos which were described in the previous sections.

### 7.2. Evaluation Results

There are two binary classes in this research. The first is for breast detection using the cascaded Adaboost and the second is for nipple detection using the neural network.

#### 7.2.1. Breast Detection

The data set is divided as in Table 1 using the holdout method. The resultant confusion matrix for the breast detection is shown in Table 2. The evaluation parameters for the breast detection are calculated and presented in Table 3. As shown in Table 4, the accuracy of the breast detection is 98.2%.

#### 7.2.2. Nipple Detection

The data set is divided as in Table 4 using holdout method. The resultant confusion matrix for the nipple detection is shown in Table 5. The evaluation parameters for the nipple detection are calculated and presented in Table 6. As shown in Table 6, the nipple detection algorithm has 92.3% accuracy and 89.0% precision.

#### 7.2.3. Nipple Tracking

The nipples are tracked using the template matching method. Three types of template matching are evaluated in this section using Dataset-3. The accuracy of the nipple tracking algorithm is computed using the* F*-score method. The evaluation matrix of nipple tracking using the normed square difference (NSDTM), normed correlation matching (NCTM), and NCCOEF is presented in Table 7. Based on the presented data in that table, the accuracies of the nipple tracking using the NSDTM, NCTM, and NCCOEF are 94.9%, 95.8%, and 97%, respectively.

Based on the evaluation of the nipple tracking methods, the normed correlation coefficient method was selected for using in the BSE evaluation algorithm. As described in the evaluation performance, the mentioned tracking method has the highest accuracy amongst other methods.

#### 7.2.4. Palpation Detection

The palpation detection algorithm is evaluated using the videos in Dataset-4. For this evaluation, only one block in the image sequences is analyzed. The resultant confusion matrix for the evaluation of palpation detection is shown in Table 8. There are 20 positive and 30 negative videos in this evaluation. Positive videos are those in which the palpation is performed correctly, whereas negative videos are those in which palpation is not performed correctly or at all.

The evaluation parameters of palpation detection are calculated and presented in Table 9. Based on the data in the confusion matrix shown in that table, the palpation detection algorithm has 92% accuracy and 86.3% precision.

#### 7.2.5. Evaluation of Integrated Algorithm

The integrated algorithm is created by combining the individual algorithms (breast detection, nipple detection and tracking, breast region tracking, and palpation detection). The input of the integrated algorithm is the image sequences that are taken by a webcam and the output is the highlighted blocks to show the palpated blocks and also an integer that shows the percentage score of palpated areas on the breast region.

The integrated algorithm is evaluated through five actual BSE performances. Thus, there are five tests on the left breast and five on the right breast. The resultant confusion matrix for the evaluation of integrated algorithm is shown in Table 10. The evaluation parameters of the integrated algorithm are calculated. As shown in Table 10, evaluation is performed by analyzing the palpated blocks in the breast regions. As mentioned previously, the matrix of the breast region has 6 blocks. During BSE performance, all the blocks must be palpated. Following palpation on each block, that block is highlighted to inform about the palpated and nonpalpated blocks. Based on the data in the confusion matrix (Table 10), the accuracy of the integrated algorithm is 93.3%.

### 7.3. Experiments

During the actual BSE procedure, the woman must sit in front of a webcam and perform the BSE. Both breasts must be visible on the screen before the start of the procedure. The application evaluates the BSE training in real-time. Thus, the woman can see which areas of the breast region were palpated and which areas must be palpated during the remainder of the procedure. It should be noted that the average execution time of BSE performances is 4 minutes in the experiments.

As mentioned previously, the integrated algorithm is created by combining the individual algorithms. The actual BSE test using the integrated algorithm was performed by 2 women in different luminance conditions. The results of the BSE performances were confirmed by the pathologist in the research team. The experimental results show that the integrated algorithm is robust in guiding and evaluating BSE performance in different luminance conditions.

The actual tests were performed using a notebook and a webcam (HD-A4Tech) in real-time with 20 fps. The notebook's characteristics are described in Table 11.

## 8. Conclusions

### 8.1. Summary

In this paper, the authors use the computer vision technique to calculate the percentage of the palpated blocks in breast self-examination. The proposed algorithm first detects the positions of breasts and nipples and then identifies the regions of breasts to be palpated. It then tracks the palpated regions through the video and determines the percentage of palpated blocks. Women can perform BSE using the stated application, a webcam, and a computer to evaluate their BSE performance in real-time. Unfortunately, there is lack of literature in the field of computer vision and BSE. Thus, the developed algorithm can be considered as the first step in this field. The algorithm has three main stages: nipple detection and tracking, breast region identification with blocks, and detecting the palpated blocks. The cascaded Adaboost classifier, integral imaging, color moments, and ANN are used for detecting the nipples. The template matching method (NCCOEF) is used to track the nipples in real-time. The second stage focuses on identifying the breast regions. A matrix is used to locate the breast region by tracking the opposite nipple. The breast region matrix has 6 enumerated blocks for palpation, and the palpation must be carried out on the stated blocks. The third stage focuses on detecting the palpated blocks in the breast region. A motion detection method, Farnebäck optical flow, is employed to detect the palpated blocks. After palpation, each block is highlighted, and the score of covering the breast region is monitored on the screen.

Experimental results have shown that the BSE evaluation algorithm in this paper provides robust performance. The nipple detection algorithm has 92.3% accuracy, the nipple tracking algorithm has 97% accuracy, the breast region detection has 98.2% accuracy, and the algorithm for detecting the palpated blocks has 92% accuracy.

One benefit of this application is that women can perform the BSE procedure in private without any human supervision. With this application they can comfortably check their own breasts to detect any undue abnormalities. However, if an abnormality is detected, this must be reported to a medical doctor who, upon confirmation of the presence of such abnormality, will proceed to carry out further clinical checks. It should be noted that the technique used in the paper is not very innovative, but it is a real application.

### 8.2. Future Work

Although an automated BSE evaluation has been developed in this paper, there are a number of areas in which future research, based on the results presented here, can be explored.

For future work, how to enhance system performance against partial occlusion should be considered. Here, the simple algorithm is used for the time of occlusion on nipples. In this study, BSE for the left and right breasts must be performed with the left and right hands, respectively. By resolving the occlusion issue, BSE can be performed with no limitation of using either the left or right hand.

In the nipple tracking section, since the target has a distinguishing color feature and is deformable, the mean-shift algorithm can be considered as an appropriate method in tracking the nipples. In the future work, the nipple tracking will be tested using the mean-shift algorithm and the results would be compared with the template matching method that was used in this paper.

The motion of the fingers on the breast regions is successfully detected using the Farnebäck method. However, to enhance the model, the circular motion of the fingers should be recognized and detected in the future work, since the circular motion of the fingers has a better result in sensing abnormalities in breast regions. The other optical flow techniques would be tested and compared with the current results and presented in the future work.

In this study, a matrix with 6 blocks is assigned to identify the breast region. While this appears to be valid, the pathologist's suggestion is that it would be advantageous if the grid is changed to a quadrant system, with the nipple as the center. This is the natural way doctors examine the breast and report findings. Thus the left and right breasts will each have four quadrants: left upper outer, left upper inner, left lower outer, and left lower inner quadrants. Another possibility would be to further subdivide the breast similar to an analog clock face, again with the nipple as the center of the clock. The breast is then divided into twelve sections as in a clock. Areas involved with abnormality would then be described as “at around 3 o'clock” or “between 8 and 9 o'clock.” This provides a common understanding and can easily be transmitted from doctor to doctor with a clear understanding of location of the mass or abnormality.

Finally, in this study, the BSE is evaluated in terms of coverage area of the entire breast region. However, detection of the degree of pressure on each block of the breast region is an important issue that should be solved in the future, since a mass or abnormality might exist at various depths of the breast.

## Figures and Tables

**Table 1:** Training, test, and validation set for breast detection.

	Training	Validation	Test
Positive images	700	50	250
Negative images	8000	500	1500

**Table 2:** The resultant confusion matrix for breast detection.

	Predicted	
	Negatives	Positives
Actual		
Negatives	1478	22
Positives	9	241

**Table 3:** Performance of breast detection classifier.

Accuracy (AC)	0.982
True positive rate or recall (TP)	0.964
False positive rate (FP)	0.014
True negative rate (TN)	0.985
False negative rate (FN)	0.036
Precision (P)	0.916

**Table 4:** Training, test, and validation set for nipple detection.

	Training	Validation	Test
Positive images	150	30	70
Negative images	5450	50	100

**Table 5:** The resultant confusion matrix for nipple detection.

	Predicted	
	Negatives	Positives
Actual		
Negatives	92	8
Positives	5	65

**Table 6:** Performance of nipple detection classifier.

Accuracy (AC)	0.923
True positive rate or recall (TP)	0.928
False positive rate (FP)	0.08
True negative rate (TN)	0.92
False negative rate (FN)	0.05
Precision (P)	0.890

**Table 7:** The evaluation matrix for nipple tracking.

	NSDTM	NCTM	NCCOEF
Number of true positives	723	736	755
Number of false Positives	77	64	45
Precision	0.903	0.92	0.943
Recall	1	1	1
*F*-score	0.949	0.958	0.970

**Table 8:** The resultant confusion matrix for palpation detection.

	Detected	
	Negatives	Positives
Actual		
Negatives	27	3
Positives	1	19

**Table 9:** Performance of palpation detection evaluation.

Accuracy (AC)	0.92
True positive rate or recall (TP)	0.95
False positive rate (FP)	0.1
True negative rate (TN)	0.9
False negative rate (FN)	0.05
Precision (P)	0.863

**Table 10:** The resultant confusion matrix for the integrated algorithm.

The block numbers on the breast region	Detected					
	1	2	3	4	5	6
Actual palpation						
1	9	0	0	0	0	1
2	0	8	0	0	2	0
3	0	0	9	1	0	0
4	0	0	0	10	0	0
5	0	0	0	0	10	0
6	0	0	0	0	0	10

**Table 11:** The notebook's characteristics for the actual tests.

Operating system	Windows 7
System type	64 bit
Processor	P 6000 @ 1.87 GHz 1.87 GHz
RAM	2.00 GB
IDE	Qt + OpenCV Library
